# Inhibition studies of HBV DNA polymerase using seed extracts of Pongamia pinnata

**DOI:** 10.6026/97320630015506

**Published:** 2019-07-31

**Authors:** Manikannan Mathayan, Selvaraj Jayaraman, Langeswaran Kulanthaivel, Arumugam Suresh

**Affiliations:** 1 Centre for Drug Discovery and Development, Col. Dr.Jeppiaar Research Park, Sathyabama Institute of Science and Technology, Chennai; 2 Department of Biochemistry, Saveetha Dental College and Hospital, Chennai 77; 3 Department of Bioinformatics, Science Campus, Alagappa University, Karaikudi

**Keywords:** DNA, HBV, polymerase, Pongamia pinnata

## Abstract

Several antiviral compounds for HBV have been identified from traditionally used medicinal plants. We have earlier described the immune modulation properties of P. pinnata, a traditionally used Indian medicinal plant. Therefore, it is of interest to explore the anti-Hepatitis B virus activity of P. pinnata extracts by in-vitro screening assays. This study clearly demonstrated that the 5mg/ml concentration of the aqueous extract significantly inhibited the virus binding. Further, the spectral study was carried out for finding active compounds. The active chalcone derivatives namely, glabaarachalcone, and isopongachromene were isolated from P. pinnata aqueous seed extracts by standard spectral procedures. Virtual screening data shows that glabaarachalcone, and isopongachromene bound with HBV DNA polymerase protein target.

## Background

Though viruses are smaller in size and appear simple by their structure, the diseases caused by viruses are complex and devastating and if untreated could be fatal. Virus infections range from milder flu like diseases to deadly hemorrhagic fevers, which are extremely fatal [1].One of the structurally simple and dreadful disease causing virus is Hepatitis B virus (HBV), which is a small circular DNA virus containing a nucleocapsid and an envelope. HBV causes jaundice and in majority of the individuals the primary infection is self-limited and no complication is noticed. However, if the infection becomes chronic then it developed as fatal disease. In India more than 37 million HBV carriers and contributes a large proportion of this HBV burden [2].

Approximately 2.57 billion people worldwide are infected by HBV and over 400 million develop chronic viral hepatitis and up to one million people die every year from the complications of HBV infections [3]. Several drugs are currently available for the treatment of chronic viral Hepatitis, which includes interferonalpha therapy and the usage of nucleoside/nucleotide analogs (lamivudine, adefovir, entecavir, telbivudine and tenofovir) [4]. Yet, treatment is limited by the adverse effects of interferons and the emergence of nucleoside/nucleotide-resistant mutants [5]. In addition, non-responder population is relatively high. These gaps necessitates to lookout for a novel anti-HBV drug from various natural sources. Indian medicinal plants have been a promising source of novel drug discovery. P. pinnata seeds and its oil have been traditionally used by Indians for treating several diseases [6]. Pongamia formulations are widely used to treat bronchitis, and this plant compounds widely studies for various biological properties, which includes diabetes [7]. Pongamia pinnata seed oil is topically applied to treat skin diseases, leprosy, rheumatism, gonorrheal ulcers and has been reported for anti HSV activity [8]. In our previous study, we reported that the seed aqueous extracts of P. pinnata's has immune modulation potential and anti HIV activity [9]. With this note, our study is further extended to investigate the anti HBV inhibition activity of this plant compounds.

## Methodology

As described in our previous report [10], P. pinnata seeds were collected and authenticated before extraction. Extracts were made at different concentrations and stored at 4°C for further analysis of its biological activities.

## HBsAg binding inhibition assay:

Hepatitis B positive plasma (HBeAg and HBsAg positive) was obtained from Voluntary Health Service (VHS) Blood bank, Adyar, Chennai. HBV titer was estimated by measuring the concentration of Hepatitis B Surface Antigen (HBsAg) in the plasma. HapanostikaHBsAg ultra ELISA kit was used for this quantification. The HBsAg titer in the HBV positive plasma and the titer of HBsAg were calculated based on the standard curve. The titrated viruses were stored at-196°C until use. The assay was performed as described in the previous reports [13,14].

For the assay, equal volume of pre-titrated HBsAg positive plasma was mixed with P. pinnata extracts and incubated at 37°C for 5 days. The mixture was assayed daily for the presence of bound/unbound HBsAg using (HapanostikaHBsAg ultra, France) ELISA kit. An in-house preparation, served as drug positive control, which inhibited more than 90% of HBV. A potent anti HIV drug Nonoxonol-9 served as negative HBV drug, which showed >1.0% activity. The ELISA was performed as per manufacture's instruction. The results are presented as percent (%) inhibition and were calculated as follows. Percent inhibition = (OD of control-OD of Test)/OD of control X 100. All assays were repeated 3 times and the result represents the average (mean) and standard deviation (SD) of 3 experiments.

## Cytotoxicity assays on P. pinnata aqueous seed extracts:

Extracts or Triton-X (Positive control for toxicity) or negative control were mixed with human HepG2 cells and incubated at 37°C for 78 hours. Then the cells were tested for their viability as a direct measure for extract induced cytotoxicity. This was done by MTT (Dimethyl trizoldi phenyl tetra solium bromide) (Sigma-Aldrich, Cat. No. M2003) assay as described before [16]. Towards this HepG2 cells were plated at a concentration of 0.5 million cells/ml in a 24 well culture plate. At the end of 72 hours the cells were harvested and treated with 250 µl of MTT solution/well and incubated at 37°C for 2 hours. Then 200 µl of DMSO was added and further incubated at 37°C for 2 hours. The plates were read at 540 nm and the percentage of viability was calculated using the formula as described previously. Cellular viability was also tested by trypan blue dye exclusion technique as described elsewhere [15].

## Phytochemical analysis of P. pinnata seed extract:

P.pinnata seed extract was subjected to preliminary phyto chemical screening as per data published in our previous paper. Based on this experiment, aqueous extracts were evaluated for the active compounds, which are possibly associated for the observed bioactivities. P. pinnata seed extracts were subjected for compound identification by injecting 1µl of extracts into the GC-MS (JEOL GC mate) instrument. After running for 40 minutes, major compounds were identified by comparing with standard references [10].

## Molecular docking of HBV DNA polymerase with chalcones:

### Protein preparation and homology modeling:

The X-ray crystallographic structure of HBV DNA polymerase protein target is not available in PDB. Protein sequence was retrieved from Genbank (Accession No.: AGA95798.1). HBV DNA polymerase has 843 amino acid residues. The protein sequence was used to develop the homology-modeled structure. The threedimensional coordinates of HBV polymerase was developed using SWISS MODEL a comparative protein-modeling program. It computes a model based on the alignment of the sequence to be modeled with known related 3D structures. Water molecules, ligands and other heteroatoms were removed from the protein molecule. Addition of hydrogen atoms to the protein was performed using CHARMm force field. Energy minimization was performed by using conjugate gradient method with an RMS gradient of 0.01kcal/Å mol on Argus lab [10, 11,12].

### Molecular Docking:

The grid-based molecular docking method is used here using the program tool from Argus lab. 4.0.1 Version, that employs the CHARMm force field. The target is held rigid while the ligands are allowed for flexible docking. The ligands were retrieved from drug bank, Canada as per GC MS results. Commercially available antiviral drugs were analyzed with the target as a control for docking. Hence, it is possible, to specify the ligand placement in the active site using a binding site sphere. Then the prepared ligands such as glabarachalcone, isopongachromene and karanijin are docked to the active site using default parameters. The results of the docking enabled the ranking of the docked conformation of the ligands according to their docking score and hydrogen-binding site. Based on standard antiviral drug, chalcones compound were selected as hits for the target protein [10, 11,12].

### Analyses and visualization of the ligand binding sites:

The docking poses were ranked according to their docking scores. The scoring function in docking score was used to predict the binding affinity of one ligand to the target molecule. In addition to the structural information, each record includes the docking score reported as negative value, where the higher value indicates a more favorable binding. This enables the energy to be used like a score. This score includes internal ligand strain energy and receptorligand interaction energy, and is used to sort the poses of each input ligand. The molecular visualizations of the docked complexes were analyzed using the Argus lab version 4.0.1. [10, 11,12].

## Results

**Figure 1:**
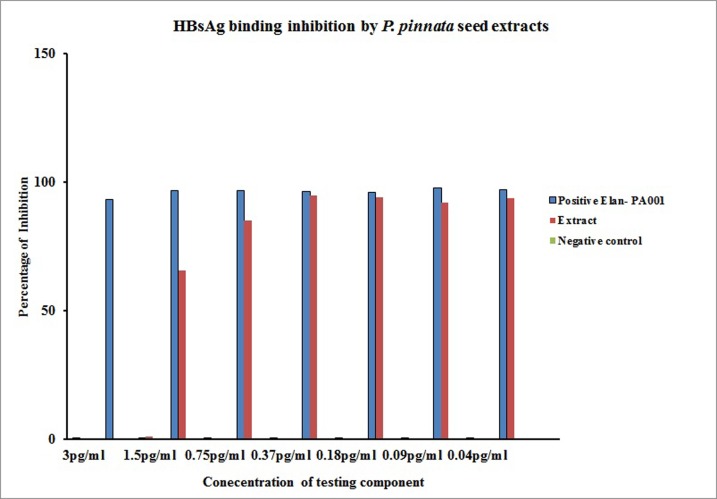
HBs Ag Binding inhibition by aqueous seed extracts of P. pinnata. 5 mg/ml concentration of P. pinnata aqueous seed extract treated with HBs Ag Positive plasma. The mixture was assayed daily for the presence of bound/unbound HBsAg using (HapnostikaHBsAg ultra, France) ELISA kit. Negative control- Nonoxynol-9 treated culture served as negative control, Elan PA001 treated as positive control.X axis- denotes percentage of inhibition Y –axis denotes HBV viral concentration present in the Positive plasma sample.

The anti HBV activity of 5 mg extract of P. pinnata seed was treated with varying concentrations of the virus (3 pg/ml up to 0.04 pg/ml) (Figure 1). For this experiment, an in-house preparation (Elan-PA001) served as a positive control and nonoxynol-9 (used as a positive control in anti HIV study only) was treated as a negative control. P. pinnata seed extract significantly inhibited the HBV at a concentration of 0.18 pg/ml (in comparison with negative control group p<0.001). However, when the virus concentration was escalated, the inhibitory effect started to diminish and at 3 pg/ml the inhibitory activity completely abrogated. Though P. pinnata seed extracts did not show HBsAg binding inhibition as that of in house positive control, its inhibitory level was much higher than that of the negative control and these values are significant.

**Figure 2:**
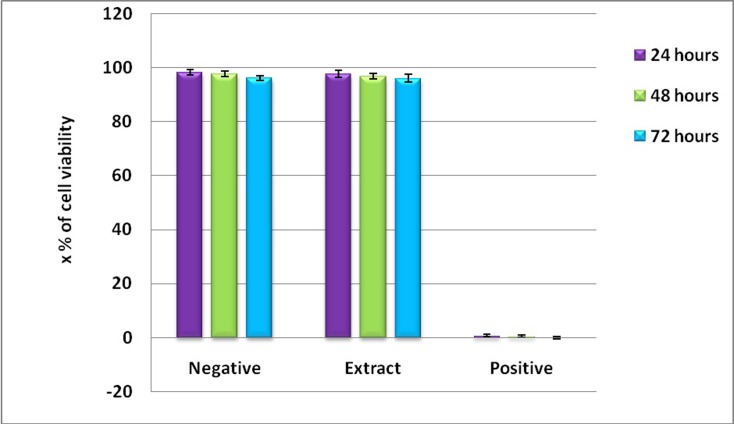
Toxicity of P. pinnata aqueous seed extracts on Hep G2 cells. 2x10^4^Hep G2 cells were stimulated with upto 5mg/ml concentration of P. pinnata aqueous seed extracts. After 72 hours of post stimulation cells were treated with 250µl of MTT solution/well and incubated at 37°C for 2 hours. Plate was read at 540nm and the percentage of viability was calculated. Cells viability was represented in percentages. Negative control- distilled water treated culture served as negative control, Triton-x-treated culture served as positive control.

In the above experiments, it was seen that P. pinnata seed extracts were found to exhibit significant anti HBs Ag activity. For all the above studies upto 5mg/ml concentration of aqueous extract was used. And hence these extracts were treated with vero cells and human PBMCs and the toxicity was evaluated by MTT assay as described in the materials and methods section. The toxicity is represented as percentage of viable cells upon extract treatment or positive control (Triton-X) or negative control (Distilled water). As shown in the Figure 2 the P. pinnata aqueous seed extracts have not shown any toxicity as revealed by 96.1 ± 1.4 percent viability upon 72 hours of post treatment. Thus, from this study it could be concluded that P. pinnata aqueous seed extracts upto 5mg/ml concentration was non-toxic. Almost similar levels of viable cells were found by trypan blue dye exclusion technique.

**Figure 3:**
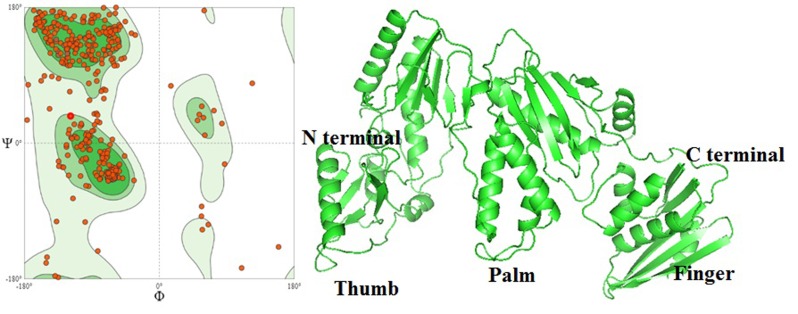
HBV DNA polymerase homology modelling a) validation of HBV polymerase by Ramachandran Plot; b) homology model of HBV DNA polymerase (AGA95798.1)

**Figure 4:**
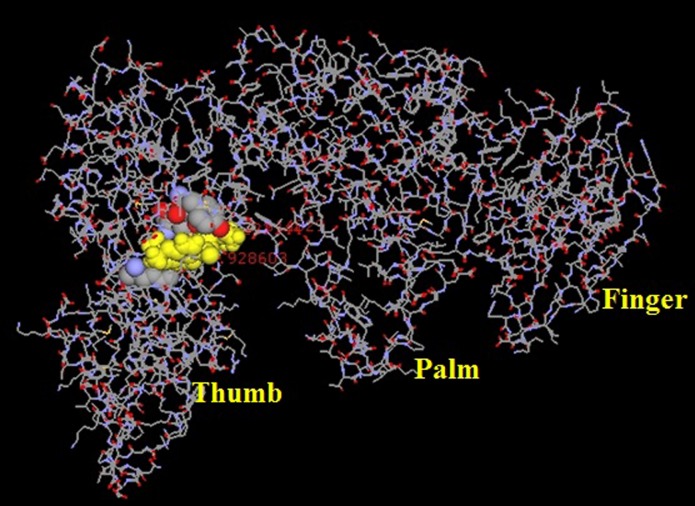
Interacting portion of the glabarachalcone ligand with Hepatitis B DNA polymerase

The HBV DNA polymerase sequence obtained from Genbank (Accession no. AGA95798.1), the tertiary structure were predicted by SWISS Model homology modeling (Figure 3). As per data obtained from GC-MS, data for the chemical structure were downloaded from drug bank and it is considered as a ligand. They docked with HBV DNA polymerase and the results clearly support the observation to be docked with Chalcones (Table 1 and Figure 4). The Chalcone docking score in the range of -8 to -9 kcal/mol is observed. Isopongachromene is the likely candidates to be considered for further studies.

## Discussion

As discussed earlier, approximately 250 million people are infected with HBV. When an individual undergo an active HBV infection, the disease follows a progressive course starting with inflammation of the liver followed by fibrosis to sclerosis and finally hepatocellular carcinoma [16]. Death due to hepato-cellular carcinoma is one of the most common forms of human cancer-related deaths [17]. HBV disease was one of the first viral diseases for which IFN therapy was shown to be effective. Interferon therapy was extremely expensive and the treatment was known to have severe side effects (fever, aching muscles and nausea) and change of behavior including suicide [14]. An important limitation for this method of treatment is the non-responder population. Later pegylated IFN has been introduced into the clinic which reduces the frequency of injections with limited success [17].

Nucleoside analogs especially acyclovir has been originally developed and evaluated for herpes viruses. Later in the 1990's a new nucleoside analog known as 1-(2'-deoxy-2'-fluoro-1-beta-Darabinofuranosyl)- 5-iodouracil (FIAU) was developed but was withdrawn shortly after because of the increased fatality by the drug. After a few years, lamivudine was found to be beneficial for about 15-30% of patients with chronic viral hepatitis and the drug was apparently safe to use. The mechanisms of lamivudine action rely upon its interaction with HBV reverse transcriptase [18]. Besides this, other drugs such as adefovir, entecavir, emtricitabine, and telbivudine have been tested either alone or in combination. A drug that can offer a 100% cure is still on the horizon. These limitations warrant a search for newer drugs including from natural source such as Pongamia pinnata. Hence, our study was focused on addressing the search for a newer anti HBs Ag inhibitory compound [19].

A wide variety of medicinal herbs have been reported to have strong antiviral activity and some of them have already been used to treat animals and people who suffer from viral infection. Aporosal indleyana and Phyllanthus amarus has been reported for having anti HBV activity by HBsAg binding inhibition assay [7, 8,10]. Chronic HBV infections can lead to liver cirrhosis and hepatocellular carcinoma. Considering the severity of clinical outcome, proper treatment modalities must be in place to fight against human HBV infection. One of the important proteins of HBV is the surface antigen (HBsAg), which helps the virus in adherence to the target tissue [14,16]. Importance of HBsAg is multifold and it is highly immunogenic. Presence of HBsAg in a patient is an indication that it is a recent infection and antibodies to HBs (anti HBs antibody) are efficient in clearing the HBV. Besides that, there are two other antigens namely HBcAg and HBeAg, which are important for the complete clearance of the virus during chronic infections. In chronic HBV infection both HBsAg and antibodies to HBs (anti HBs) are found in the patients and presence of HBsAg helps in the new infection of hapatocytes [13].

HBV infection has been effectively controlled by Phyllanthus amarus (P. amarus). In a study conducted by Thyagarajanet al.(1988), it was found that a vast majority of HBV chronic carriers (59%) have cleared HBV as revealed by the loss of HBsAg within 15-20 days of P. amarus treatment and stayed HBsAg negative for about 9 months [20,21]. In the present study, anti HBV activity of P. pinnata seed extract was performed. As shown in the Figure 1 P. pinnata extract at a concentration of 5 mg/ml showed an inhibition of 85% of HBV (virus concentration of 0.75 pg/ml), which is a remarkable amount of viral suppression. Inhibition of HBV started to diminish when the virus concentration was increased to 1.5 pg/ml and above which suggest that further tune-up is needed. It is important to record that anti HBV activity was noticed only with aqueous extract and not with other extracts (methanol, ethanol, etc.-data not shown here) which may be an indication that there could be other solvents that needs to be further explored [20,21,22].

HBV infects hepatocytes and causes viral hepatitis. Receptors for HBV is not fully known and it is speculated that preS domain of surface protein of the virus bind to carboxypeptidase D molecules found on hepatocytes. In the ELISA plate that was used for coating antibody was against small S protein of HBV. This monoclonal antibody 4-7B is specific for residues 178-186 in the small S protein (S-HBs) with the amino acid sequence PFVQWFVGL. This is the antibody with which the ELISA plate was coated with. S-HBs (also known as major protein) are the most abundant and known for its viral binding activity. So, the interference of P. pinnata could be at the level of s-HBs of HBV. Further experiments are needed to support this hypothesis. Thus, the surface antigen (HBsAg) plays an important role in virus attachment to the hepatocytes and any methodology that would interfere with this initial binding can prevent the virus attachment to the host tissue. In this context, the current investigation is very important. Hence, the study shows P. pinnata extract inhibit HBsAg binding to its receptor and in this study anti HBs antibody act as the receptor. This study demonstrated that 5 mg/ml concentration of the extract inhibited the virus binding and this inhibition was noticed up to 0.75 pg/ml concentration of the virus. This is the first report to show that P. pinnata extracts are very efficacious in inhibition of HBV binding to its receptor. This study also opens up new avenues to explore the molecular mechanisms of HBV viral entry inhibition. Thus, P. pinnata has a wide scope to use it as medicine against infectious diseases. In the study, the observed inhibition could be due to the interference with small S protein of HBV as monoclonal antibody. To this epitope was used as coating antibody in the ELISA kit. This monoclonal antibody 4-7B is specific for residues 178-186 in the small S protein (S-HBs) with the amino acid sequence PFVQWFVGL and it is used for coating the ELISA plate. S-HBs (also known as major protein) are the most abundant and it is known for its viral binding activity. It is possible that HBV entry could be through a multifold mechanism and more than one region within HBsAg is possible.

Pongamia have long been used in traditional oriental medicine for treatment of skin infections. Inhibition of viral DNA polymerase (reverse transcriptase) upon P. pinnata treatment has not been tested in HBV model. As per data obtained from GC-MS, chalcones chemical structure was downloaded from drug bank. Chalcones are considered as a ligand and they docked with HBV DNA polymerase (Table 1). The two ligands had the docking score in the range between -8 to -9 kcal/mol with HBV DNA Polymerase. Since glabarachalcone and isoponga chromene are showing a higher negative value of docking score compared with other compounds they would be the likely candidates to be selected for further studies. In support of the previous analyses and results, isopongachromene integrated with HBV DNA polymerase by making four H-bonds with a distance <2.8 Å; among them, H-bond interaction with 349LYS, 346GLU, 392LYS, 348PHE. Similarly, glabarachalcone interacted HBV DNA Pol making three H-bonds such as 117TYR, 185TYR and 163GLN (Figure 4). It is of further interest to find out the common motifs/residues in HBV that interact with two ligands. Thus, this analysis strongly supports the earlier observation that glabarachalcone and isopongachromeneare the best possible candidates that can be taken up further in search of a drug candidate.

## Conclusion

HBsAg binding inhibition assay is an in vitro assay to find agents with anti HBV activity. This method can reveal if the drug has any interference with HBsAg binding to its receptor. In vitro study shows that P. pinnata seed extract interfered with HBsAg and thus probably may prevent HBV entry. However, its inhibitory activity was noted only with lower virus dose and this inhibition was abrogated if the virus concentration was increased. In Silico screening study shows that glabarachalcone and isopongachromene are potential ligands for HBV DNA polymerase target. However, further optimization is required by in vivo studies for evaluation. This is the first report to show that P. pinnata extracts are very efficacious in the inhibition of HBV binding to its receptor. This study also opens up new avenues to further explore the molecular mechanisms of HBV viral entry. Thus, P. pinnata has major application as a medicine against infectious diseases. The assay used here is a cell free system and thus cell based assays need to be used to improve its efficiency. HBV replicon based transgenic animal models are available and these models could be used to further optimize drug's effect in in vivo situations. Non-human primate models are alternative methodology to study the in vivo efficiency. Finally P. pinnata's efficiency needs to be tested in clinical trials. Though it appears to be a long way to take it to clinical practice, it needs to be reiterated that this study is an eye opener to pave way for future research.

## Figures and Tables

**Table 1:** Docking scores of Chalcone derivative against HBV DNA Polymerase targeted protein

Ligand Name	Hydrogen Bond	Score	Kcal/mol	Amino acid positions	Distance
Zidovudine	8	-9.71349	-7.9263	68Lys	2.89
				69ASP	2.92
				191LEU	2.77
				222Lys	2.17
				222Lys	2.256
				112ASP	2.609
				224GLN	2.99
				230LEU	2.853
Isopongachromene	4	-9.094	-8.78372	349LYS	2.74
				346GLU	2.85
				392LYS	2.45
				348PHE	2.28
Glabaarachalcone	3	-9.52387	-11.3069	117TYR	2.92
				185TYR	2.61
				163GLN	2.22
